# Artificial neural networks applied for predicting and explaining the education level of Twitter users

**DOI:** 10.1007/s13278-021-00832-1

**Published:** 2021-11-01

**Authors:** Alexandru Razvan Florea, Monica Roman

**Affiliations:** 1 Bucharest University of Economic Studies, Bucharest, Romania; 2 IZA Bonn and CELSI Bratislava, Bucharest University of Economic Studies, Bucharest, Romania

**Keywords:** Artificial neural networks, Social network, Deep learning, Face recognition, Data collection, RStudio

## Abstract

This paper provides a novel procedure to estimate the education level of social network (SN) users by leveraging artificial neural networks (ANN). Additionally, it provides a robust methodology to extract explanatory insights from ANN models. It also contributes to the study of socio-demographic phenomena by utilizing less explored data sources, such as social media. It proposes Twitter data as an alternative data source for in-depth social studies, and ANN for complex patterns recognition. Moreover, cutting edge technology, such as face recognition, on social media data are applied to explain the social characteristics of country-specific users. We use nine variables and three hidden layers of neurons to identify high-skilled users. The resulted model describes well the level of education by correctly estimating it with an accuracy of 95% on the training set and an accuracy of 92% on a testing set. Approximately 30% of the analyzed users are highly skilled and this share does not differ among the two genders. However, it tends to be lower among users younger than 30 years old.

**Supplementary Information:**

The online version contains supplementary material available at 10.1007/s13278-021-00832-1.

## Introduction

Over the past four decades, as computer power and mobile devices impact human activities, more and more people use virtual environments for social interactions such as communication, learning, meeting new people, getting the news, and many more. Social Networks (SN) platforms passed the volatile days, and the possibility of instantly connecting people from all over the world became a certainty in less than two decades. Several platforms, such as Facebook, Instagram or Twitter are among the most preferred ones (Perrin 2015). Twitter is an online network platform on which users post short pieces of information known as "tweets". It is also accessible by anyone, which makes it one of the most flourishing communication platforms.

There are various studies using social media data that explore social behaviors such as human movement, peace and happiness, cultural understanding or complex indicators about the quality of human life, like health status (Culotta et al. 2015; Mitchell et al. 2013; Murthy et al. 2016). However, there is a limited number of studies centered on understanding the community of social media users and their characteristics in emerging countries, such as Romania.

This paper aims to analyze the SN users’ level of education by employing advanced statistical techniques and tools like Artificial Neural Networks (ANN) and R programming language. Using unstructured data, we aim at predicting and explaining the skill level, in a case study focused on the Twitter users in Romania. A high-skilled person is defined in this paper as a person with tertiary education level or a person who owns a position in a company that usually requires tertiary education.

The novelty of the paper is four-fold: (i) from a methodological perspective, we test the adequacy of using ANN for describing population social characteristics, such as education level in unstructured datasets; (ii) we propose an innovative method to assess the impact of the input-variables on the final prediction; (iii) we focus on measuring the level of education for each Twitter user starting from the words he/she used to describe himself/herself. Our research considers new factors related to the complexity of the phrases, expressed by the average length of words and by the average number of words per tweet; (iv) we use Romanian Twitter users as a case study, filling a gap in the existing literature of ANN and SN applications.

We also include in our methodology various data mining techniques, such as deep learning algorithms for face recognition (Florea and Roman 2018a, b, c). We rely on the R programming language for data collection and statistical analysis (Florea and Roman 2018a, b, c).

The paper is structured in five main sections. The next section briefly introduces the relevant applications of SN and ANN and argues the role of the current research. The third part explains how Twitter data can be collected, details the methodology applied in the paper and describes the dataset and the demographics of the Romanian Twitter users. In the fourth section, we describe the results and we differentiate the high-skilled users from non-high-skilled users. The final section provides a review of the main outcomes, research limitations and potential next steps for future research.

## Literature review

The applications in the area of Social Networks and Artificial Neural Networks, as modern and flexible tools used in social sciences and economics, are emerging. To our knowledge, there is no evidence of the ANN being leveraged to estimate the level of education in the case of Twitter users, from Romania or elsewhere. Also, the literature on applying an ANN-based approach to SN data is still rather limited in spite of the obvious research potential of such applications (Le et al. 2016). The two subsections below briefly describe the existing results relevant for our research purpose.

### Applications of social network data in social sciences

There is no doubt that the tremendous amount of information encrypted in the billions of real-time social media interactions represents one of the most significant analytical opportunity for social behaviors (MacEachren et al. 2011; Florea and Roman 2018a, b, c). However, despite the tremendous amount of data that SN and Twitter contain, these datasets are not representative of the whole population yet. On the other hand, Twitter data can be used as a proxy for local social patterns such as human mobility (Hawelka et al. 2014; Zagheni et al. 2014). (Culotta et al. 2015). Twitter data has also been leveraged to explain the behavior of marginalized communities or even the cultures of several ethnic groups (Murthy et al. 2016).

Moreover, Twitter data were also combined with different data sources in a very original approach to measure the happiness or even obesity at the territorial level (Mitchell et al. 2013; Lerman et al. 2018).

Twitter data is also applied in the Health Sector with more and more researches receiving funds to develop new ways to use these datasets (Sinnenberg et al. 2017; Sinnenberg et al. 2016). More and more research fields, such as election forecasts, marketing, human migration or demand planning, have already adopted the use of Twitter data (Sanders et al. 2016; Luo et al. 2017).

The tremendous complexity of the social network data combined with creativity and powerful tools may lead to astonishing estimations. Researches have shown that longer words such as ‘software’, ‘development’ correlate strongly with high-skilled workers, while the ones that correlate negatively with education are generally shorter, most of the times not longer than two syllables (Mitchell et al. 2013).

### Applications of artificial neural networks

Artificial neural networks (ANN) is a machine learning modeling technique that has become considerably competitive to traditional regression and statistical models. ANN is largely used because of its exceptional performance in self-learning, adaptivity, and nonlinearity (Wang et al. 2018).

In 1943, Warren McCulloch and Walter Pitts wrote the first paper that describes how a network of neurons can be used to model simple electrical circuits (McCulloch and Pitts 1943). A few years later, Donald Hebb reinforced the concept of neurons and noticed that the neural pathways are strengthened each time they are used (Hebb 1949). The study of neural network continued in the next years involving numerous scientists like Nathaniel Rochester who led the first efforts to simulate neural networks and John von Neuman who suggested imitating simple neuron functions by telegraph relays or vacuum tubes. Nathaniel Rochester was a computer scientist who designed the IBM 701 (IBM’s first commercial scientific computer), wrote the first assembler (programming language), and participated in the founding of the artificial intelligence field. John von Neumann was a Hungarian-American mathematician, physicist, computer scientist, engineer and polymath. It was not until 1958 when Frank Rosenblatt started the work on the perceptron concept (Rosenblatt 1962). The perceptron is the oldest neural network that is still in use today. Shortly after that, Bernard Widrow and Marcian Hoff developed the first neural network (*ADALINE*) applied to a real-world problem. The names of ADALINE and MADALINE come from their use of Multiple ADAptive LINEar Elements. ADALINE was developed to identify binary patterns so that if it was able to predict the bit of a phone line by reading previous streaming bits. MADALINE was the first neural network applied to a real-world problem, being able to eliminate echoes on phone lines. In 1969, Marvin Minsky and Seymour Papert proved the Perceptron was limited (Minsky et al. 2017). The research of neural networks continued in the following years and various methodologies were published until late 1990s (Hochreiter and Schmidhuber 1997; Anon 1998).

In the past 20 years, the processing power has unlocked the potential of the neural networks enabling researches to implement ANN in various fields, such as banking (Dima and Vasilache 2016), medicine, petroleum exploration, or business decisions (Boyacioglu et al. 2009; Amato et al. 2013; Rahmanifard and Plaksina 2019; Araque et al. 2017).

ANN-based models and Twitter data have unlocked new analytical opportunities for various fields, from predicting urban traffic events (Aniekan et al. 2021) to detecting bullying behavior (Saima et al. 2021).

Twitter is one of the most widespread social networking platforms. Unfortunately, due to its vast popularity, it has also become a target for spammers. Until now, researchers have developed many ANN-based methods for identifying spammers on Twitter (Zulfikar et al. 2020).

The collection and examination of social media have also become a valuable tool for studying users' mental health and behavior tendencies (Joseph et al. 2020). Recently, artificial intelligence-based approaches have been used to study public sentiments on social media in the United Kingdom and the United States toward COVID-19 vaccines to understand the public position and concerns about COVID-19 vaccines (Amir et al. 2021). Twitter data is leveraged in a numerous public-health domains, from detecting certain events to tracking & forecasting disease evolution and even extracting geographical insights (Oduwa et al. 2020).

At the same time, the detection of cyberbullying and cyber-harassment had become a required field for social network platforms. Cyberbullying refers to aggressive behavior with offensive comments meant to harm other individuals on social media. Nevertheless, human moderation is slow and expensive, and it is not always feasible; most of the time, ANN-based models are required to detect and isolate these harmful comments and behaviors (Saima et al. 2021).

In the next section, we present how we combined applied the powerful Artificial Neural Networks to Twitter data in order to extract valuable insights about human mobility.

## Data collected and methodological approach

This section introduces the details of the non-conventional dataset utilized in this inquiry, describes the variables used in the final ANN model and briefly presents the theoretical principles and application of the ANN.

### Data collection and data description

Our research relies on R, RStudio, and Microsoft Office for the data collection, statistical tests, and visualization components. R is a widely applied programming language and an outstanding environment for producing graphs or analytical computing; it was used in this paper for subtracting the data from the Twitter database and for data analysis. R is an open-source software environment designed for statistical computing and graphics. The link between the local R environment and Twitter database is established by utilizing the official Twitter Application programming interface (API). An API is a collection of protocols and routines that facilitate the communication among multiple software engines.

For the data collection, we used an algorithm structured in 10 steps which has been previously developed and tested by the authors. It is described in our previous publications (Florea and Roman 2018a, 2019, b, c; Florea 2020) and included in the Appendix.

In the data collection process, it was used the Azure facial recognition procedure to detect and assess the demographics of Romanian Twitter users. Face Recognition Technology (FRT) has vast applicability as it can bring its contribution in various fields. FRT is already implemented in the process of matching real-time images with passports photos. Innovative studies leveraged FRT to estimate demographic characteristics starting from the available photos provided by various databases (Florea and Roman 2018a, b, c). In 2015, the FRT techniques achieved a new accuracy record of 99.65% on Labeled Faces in the Wild (LFW) data (Schroff et al. 2015).

We have collected and processed relevant data for a final sample of 3.253 unique users. (Florea 2020). The data set was retrieved in March 2020. More than 65% of the collected users are men and tend to be slightly older than the women users. The average age among women is 30 years old, while the average age of men is 35 years old. There are no significant differences across regions in terms of the user's age with the average varying between 30 and 35 in all major socio-economic regions, defined by Eurostat as NUTS-1 (Nomenclature of territorial units for statistics-1).^1^ Eurostat is the statistical office of the European Union. The highest concentration of Twitter users is in the capital of the country (Bucharest-Ilfov region). Bucharest-Ilfov and West are the regions with the most users, counting more than 45% of the total dataset. However, it is crucial to understand that even if there are regional variations in terms of popularity and engagement, Twitter is utilized as an active social media environment across all Romanian regions. The details of the dataset have been partially presented in our previous research. (Florea 2020; Florea and Roman 2019).

Online social platforms arguably represent the most comprehensive observational instrument for social behavior nowadays. Although many social platforms benefit from massive datasets, Twitter is easily accessible by social scientists offering a robust connection for their public data. However, we recognize that there are technological and legal constraints regarding the use of this kind of data (McCormick et al. 2017). The EU General Data Protection Regulation (GDPR) is the most important change in data privacy regulation in the last 20 years. We acknowledge all the legal and ethical implications in our analysis, and no data are stored at the user level.

### Artificial neural network: theoretical principles

ANN is becoming the main data analysis instrument of many industries due to their capacity to replicate the brain’s abilities. This includes using incomplete or unstructured data and making sense of it by identifying underlying patterns and similarities. It also includes the ability to generate new ideas by reorganizing existing information. As SN data is unstructured, ANN seems to be an appropriate technique for analyzing such information.

**Fig. 1:**
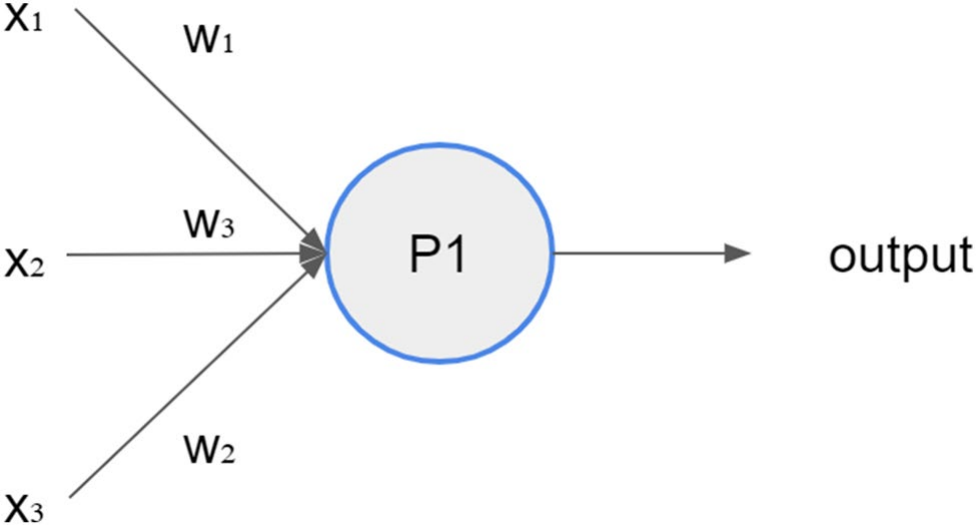
Theoretical perceptron: a neural network unit (an artificial neuron)

The first paper that described what we call today "Artificial Neural Networks" was published a few decades ago (Rosenblatt 1962). Perceptron neurons, an essential component of ANN, were developed by Frank Rosenblatt back in the 1950s and 1960s. As described in Fig. 1, a Perceptron neuron uses several binary inputs and produces a single binary output. In the above example, the perceptron P1 uses the inputs *x*_1_, *x*_2_, *x*_3_ to compile the output. The natural and effective way Frank Rosenblatt thought about this problem was to allocate weights (*w*_1_, *w*_2_, *w*_3_) to each of the factors *x*_1_, *x*_2_, *x*_3_. The output, 0 or 1, is determined by whether the weighted sum of (*w*_*j*_ * *x*_*j*_) is smaller than or greater than the threshold value.

**Fig. 2:**
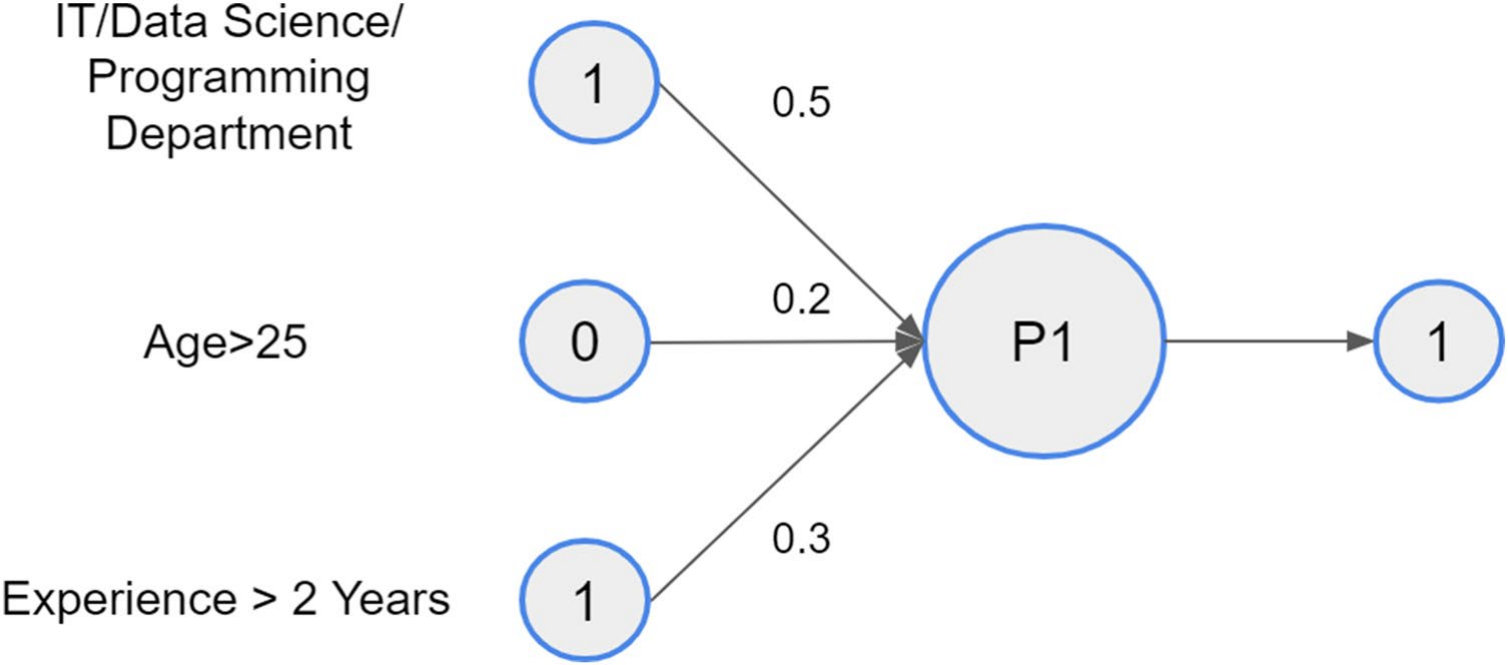
Example of a perceptron

For instance, (and in the context of this paper) we want to assess whether a person is highly skilled or not. There may be three factors of interest: *x*_1_—the person working in a department that requires tertiary education; *x*_2_—the person older than 25 and *x*_3_—the person have an experience of more than 2 years in the field. One way of estimating the level of education is by allocating weights to the three known factors: *w*_1_ = 0.5, *w*_2_ = 0.2, and *w*_3_ = 0.3. Assuming our threshold is 0.5 and we know the person works in the “Development Department” for more than 2 years and he/she is not older than 25, Fig. 2 indicates that our person is highly skilled.”

Following the methodology described above, the sum of inputs’ weights is 0.8. As this sum is bigger than the threshold (0.5), the final output is estimated to be 1.

**Fig. 3:**
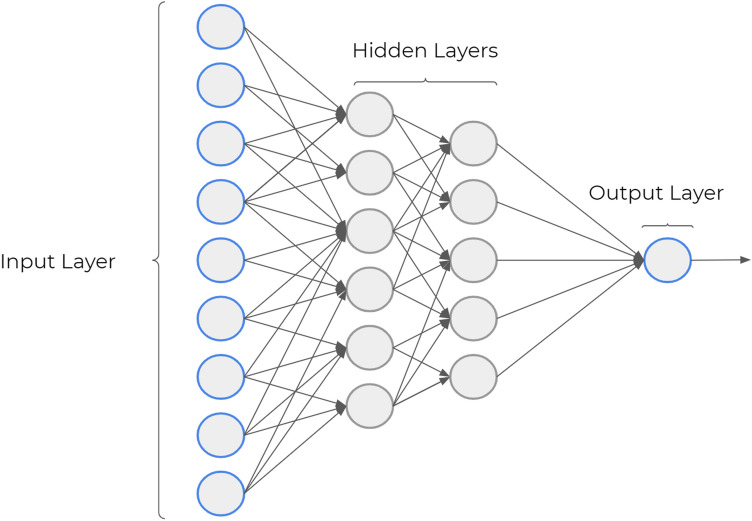
Neural network structure

The perceptron is not close to a model of human decision-making. However, it explains how a Perceptron neuron can weigh up several factors to estimate a decision. Moreover, a complex network with multiple layers of Perceptron neurons could lead to complex decisions/estimations. As described in Fig. 3, some layers of neurons are hidden to simplify the architecture of the neural network.

An essential aspect of the Perceptron is that a small variation in the weights or bias of any single neuron can make its output to flip from 0 to 1. That makes it challenging to understand how to gradually adjust the weights and biases so that the network system gets closer to the desired behavior. This problem can be solved by introducing the sigmoid neurons. Small changes in the weights of the Sigmoid neurons lead only to a small change in their output. Similarly, to Perceptron neuron, the sigmoid neuron has inputs and weights. However, the inputs are no longer restricted to only 0 or 1 as they are in the case of Perceptron neurons. Sigmoid neuron's inputs can also take any values between 0 and 1. Another crucial aspect is that the output is no longer restricted only to 0 or 1. The output is equal to σ(w*x + b), where σ is the sigmoid function described below:

1 $$\sigma \left( z \right) = \frac{1}{{1 + e^{ - z} }}$$

This particularity is vital in cases where the output cannot quantify as 0 or 1 (e.g., image processing- the intensity of the pixels in an image). After setting the right architecture of the neural network, the weights and biases are repeatedly adjusted to produce better and better outputs so that network gets closer to the desired behavior.

The brief description of the principles of the ANN show its fundamental advantages over conventional statistical models when both are compared. In ANN models, there are no assumptions on the data distribution or properties, which gives ANN a significant advantage in practical application. Moreover, unlike some traditional statistical models that require a specific set of hypotheses for testing, ANN models do not need to validate any hypotheses. They can handle fragmented data, nonlinear problems, and noise. ANN is an information management model that is similar to the biological nervous systems of the human brain.

These provide good arguments for considering ANN as an effective tool in estimating the characteristics of a population and clustering the population of Romania Twitter users into classes according to their skills. Therefore, by combining the social network platforms with advanced statistical techniques, such as ANN, we manage to reorganize the information encrypted in the social network interactions and extract novel insights from a non-conventional dataset.

### Methodological approach and variables used

The methodology applied in this paper takes the following stages: (i) We first identify the users who expressed their level of education by mentioning their current profession/role in a company (e.g., "Software Engineer at Company Name SA"). (ii) we develop and test an ANN-based model capable of differentiating high-skilled users from non-high-skilled users. (iii) we apply the model for all users and build the overall picture regarding the level of education among the Romanian Twitter users. (iv) we assess the importance of each input of the ANN analysis.

A high-skilled person is defined in this paper as a person with a tertiary education level or a person who owns a position in a company that usually requires tertiary education, as specified in their personal Twitter account description.

The neural network model proposed in this paper leverages users’ writing skills and vocabulary by analyzing their Twitter description. Following similar research (Mitchell et al. 2013), we considered the average length of words abbreviated as “avg_l_words” and the number of words abbreviated as “no_words”, as indicators suggesting the education level.

The model also uses the NUTS-1 (Nomenclature of territorial units for statistics-1) level location details of the user as the level of education may also be affected by the environment and the development of the region where individuals live. We have considered the standard socio- demographic characteristics such as gender and age, as these may bring slightly more context into our model. FRT was used in this respect, as in many cases age or gender were not explicitly provided by the Twitter users. It is important to emphasize that all users with the estimated age lower than 20 years old were removed from this data set as the graduation of tertiary education level usually happens after this age and therefore these users are not part of this exercise’s scope. Therefore, the lower age limit in our sample is 20 years old.

The next set of variables considers the popularity of Twitter users. Previous inquiries have shown that Dunbar’s Number could be successfully leveraged to measure valuable social characteristics, such as popularity (Florea 2020). Dunbar's number is a proposed cognitive limit to the number of persons with whom one can maintain stable social relationships (Dunbar 1992). This paper relies on the prior findings and partially use the indicators of popularity developed in the previous researches (Florea 2020; Florea and Roman 2019). Robin Dunbar proved that a person could maintain a maximum of 150 stable relationships (MacCarron et al. 2016; Gonçalves et al. 2011; Dunbar et al. 2012). In one of his presentations (TEDxObserver—Robin Dunbar—“Can the internet buy you more friends?”, 2012), he also indicated that, when it comes to social platforms, the limit should be adjusted to 120–130, as not 100% of the world population is present in the virtual environment. Consequently, we flagged everyone with more than 100 friends as a person with high Social Activity. Dunbar claims that the next layer of friendship extends up to 500 less stable relationships. Therefore, everyone with more than 500 followers is flagged as a popular Twitter user. Ultimately, we include the type of the profile image, as formal or informal. We defined the informal profile picture as one in which the user laughs.

Based on this set of multiple factors, the research intends to assess the level of education among Romanian Twitter users, by applying ANN-based techniques on Romanian Twitter data and to further explain the importance of each factor.

In many studies, ANNs have been confirmed to exhibit higher predictive power compared to traditional approaches (Mitrea et al. 2009; Veerachai et al. 2011). On the other hand, they have also been labeled “black boxes” due to the limited explanatory insights regarding the relative influence of the independent variables in the prediction process. (Julian and Donald 2002). Several approaches and methods were tested in the past, but none was stable enough to be widely applied (Muriel et al. 2003).

In this paper, we suggest a novel and robust approach that allows for measuring the relevance of each factor/input of the model. The method may be applied for a big variety of ANN models and provides vital explanatory insights. Our approach consists of 5 steps: (1) Train an ANN model; (2) Alter all the values of one input-variable; (3) Track the variation of the model’s accuracy; (4) Reiterate the same procedure for the rest of the input-variables; (5) Assess the relative impact of the variables.

To make sure we capture the total impact of one variable as good as possible, we are altering the values to maximize the impact on the accuracy. For binary variables, we change one into zero and zero into one. For continues variables, we first compute the minimum, the maximum, and the median. We then initialize the values below (and equal) the median with the maximum value and the values above the median with the minimum value.

For assessing the relevance of each input, we develop coefficients assigned for each input, labeled as the “relative importance coefficients” (RIC). The “relative importance coefficients” (RIC) were obtained by computing the percentage impact of each variable out of the summed impact of all variables.

2 $${\text{RIC}}_{i} = \frac{{I_{i} }}{{\mathop \sum \nolimits_{j = 1}^{n} (I_{j} )}}$$

where “RIC_*i*_” is the relative importance coefficient of the input-variable “*i*”, “*I*_*i*_” is the impact created by altering the input-variable “*i*”, “*n*” is the number of all input-variables. A higher RIC will correspond to a higher importance of the input in the ANN model.

Estimating the importance of each input-variable for an ANN model is a big step forward, as the indicator allows for identifying the variable that contribute the most to the outcome of the ANN model. However, we aim to deep dive even more into explaining how each input-variable impacts the final output. This complex question is answered by running simulations with altered data sets where all values of one input-variable are replaced with a specific value while the rest of the input-variables remain unchanged. For consistency, we only use the range of the values that were present in the training process. For example, to understand how “the average length of words” (avg_l_word) variable influences the final output (has or does not have a position which requires tertiary education), we systematically run simulations where all the observations of the analyzed variable have the same value. To be more precise, during the first simulation we initialize all the values of the analyzed variable (avg_l_word) with the lowest value the model has seen in the range of that particular variable, in our case is 0. For the second simulation, we initialize all the values of the analyzed variable (avg_l_word) with the second-lowest value. We continue the procedure for all the unique values of the analyzed variable. We finally explore the results and explain at which level of each input-variable, the likelihood of an individual to be highly skilled is maximized.

Following the described methodology, we are able not only to identify the high-skilled Twitter users and describe their profile from an unstructured dataset, but, most importantly, we can provide valuable input regarding what are the factors that impact the most the estimated education level.

## Results and discussion

For identifying the appropriate number of layers used in the ANN model, numerous models’ specifications were tested, in an iterative process, starting with one layer. We concluded that an ANN model with three hidden layers of neurons and nine input variables is robust enough for this exercise as the obtained model describes very well the level of education by estimating it correctly with an accuracy of 95% on the training dataset. Moreover, it has a good predictive power managing to predict the level of education on a testing sample with an accuracy of 92% and an area under the curve (AUC) of 0.86. The ANN model uses 80% of the initial datapoints as a training dataset and the remaining 20% of the datapoints as a testing dataset.

**Fig. 4:**
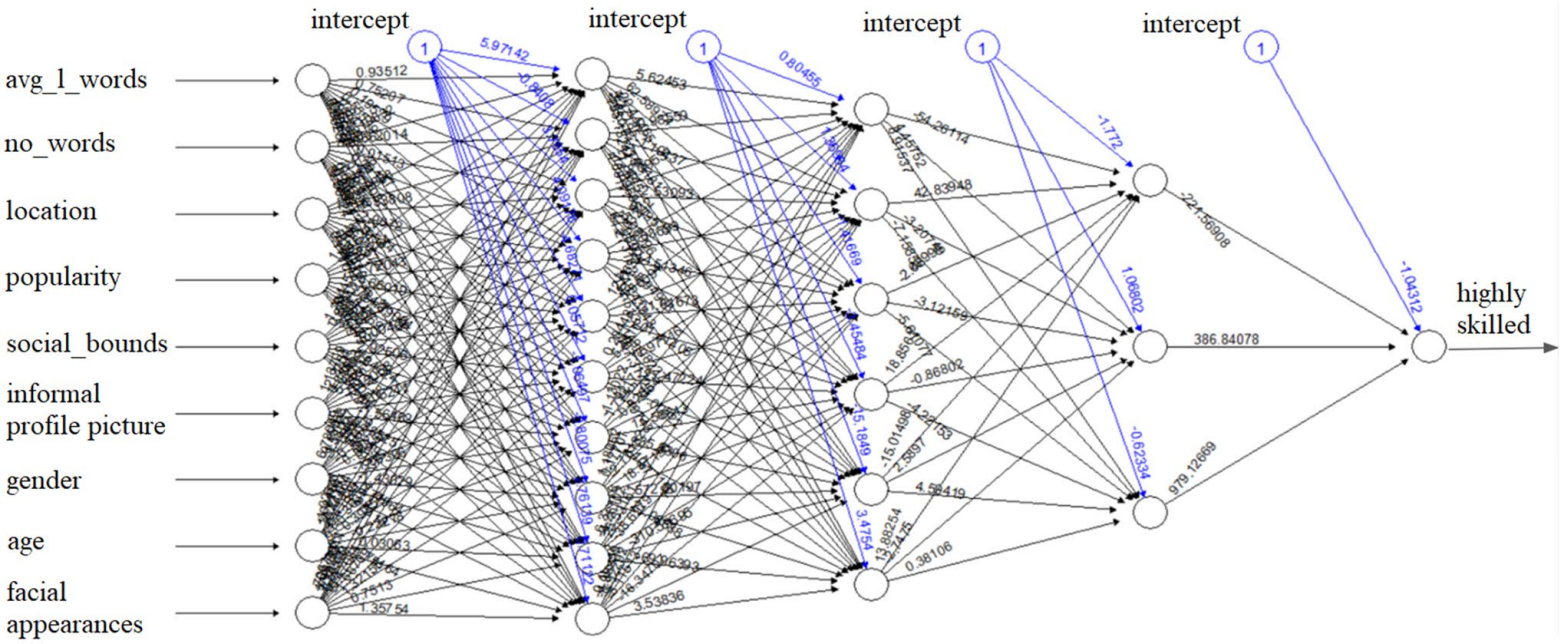
Artificial neural network model

The three layers and the factors included in the final model are presented in Fig. 4. The “average length of words”, “number of words”, and “informal picture” are critical factors for measuring the education level as they may describe the background of the user.

Moreover, the proposed model leverages demographic data, such as age, gender, and location to fine tune the results. Finally, we included variables like “the level of popularity”,” the number of social bounds”, and “facial appearances” to improve the ANN model by offering useful social media context.

**Fig. 5:**
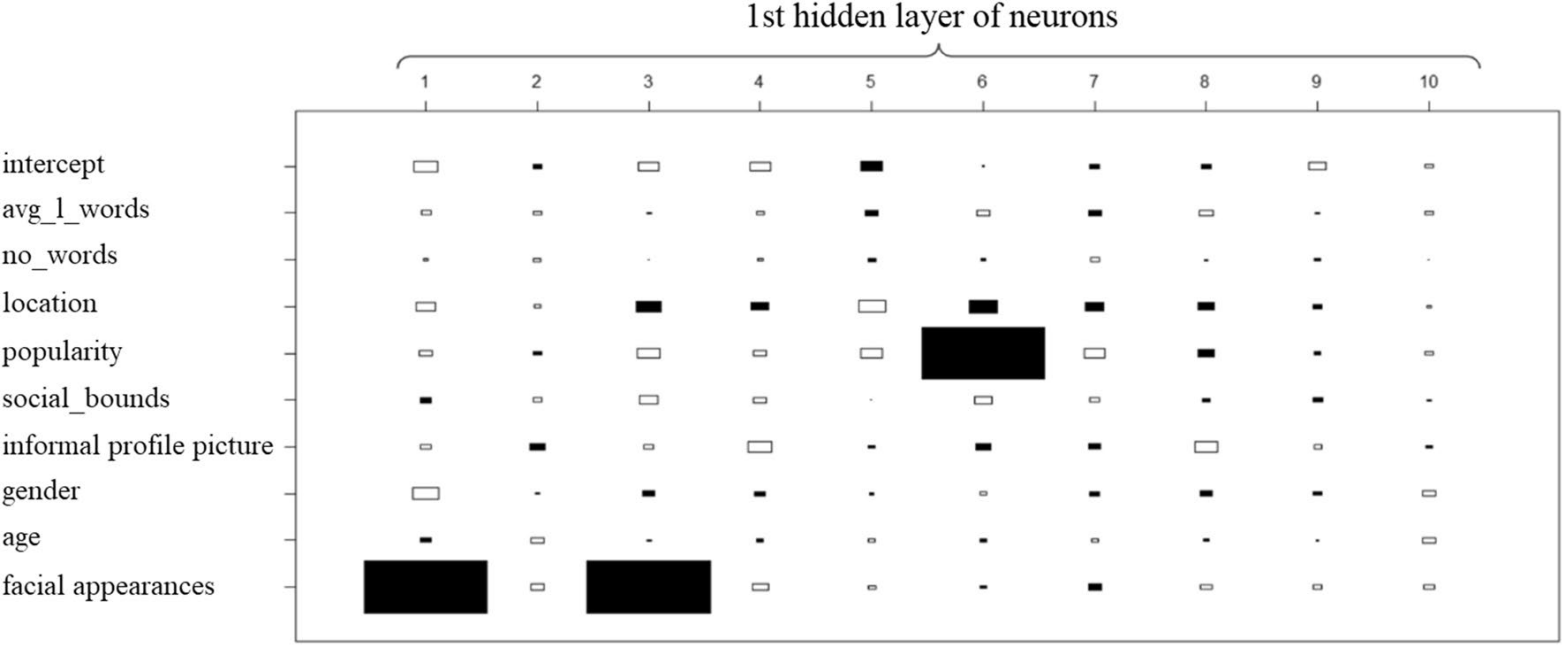
Weights for the 1st layer of hidden neurons

**Fig. 6:**
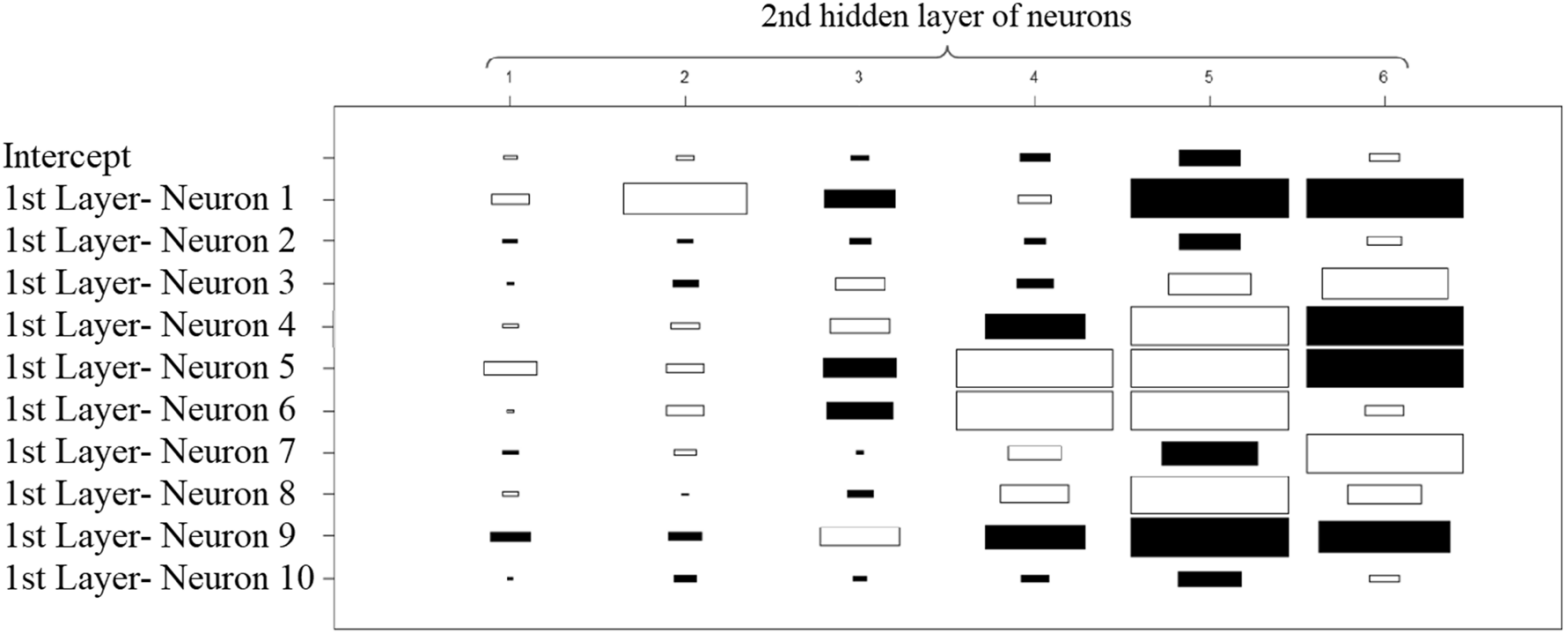
Weights for the 2nd layer of hidden neurons

**Fig. 7:**
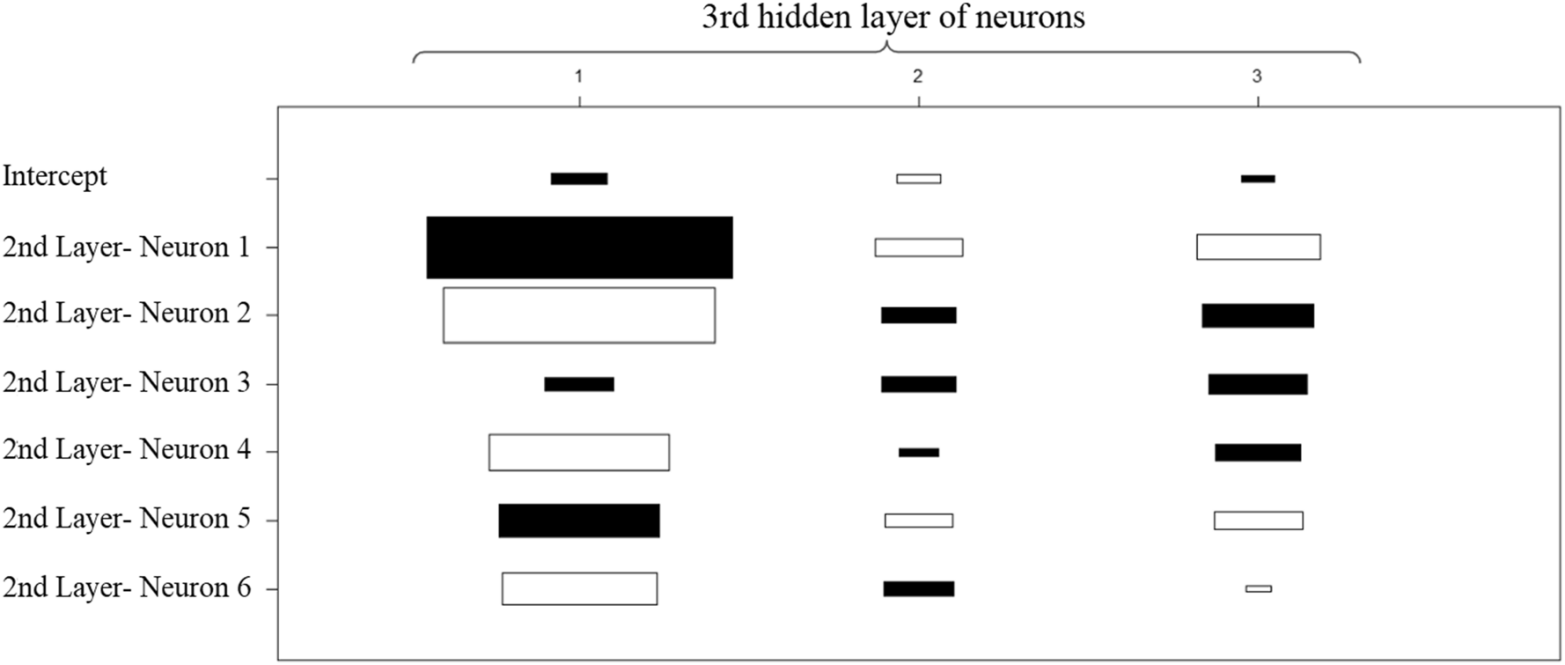
Weights for the 3rd layer of hidden neurons

**Fig. 8:**
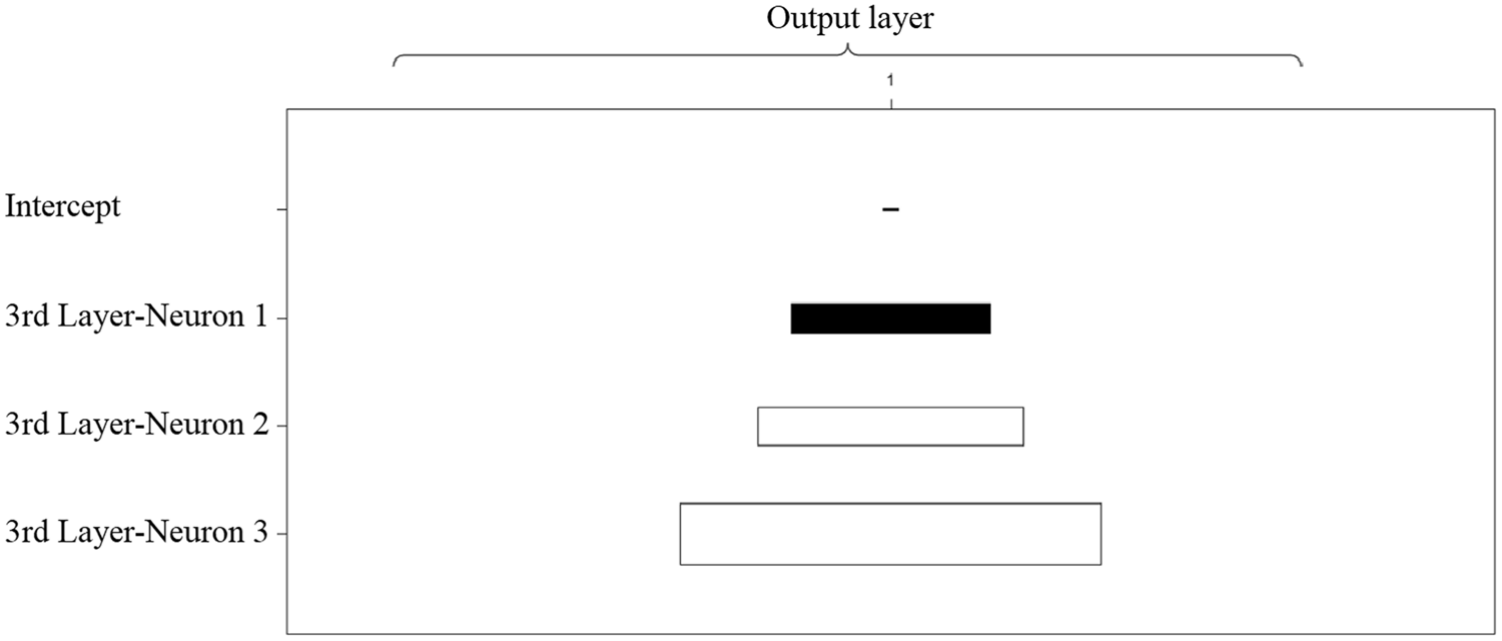
Weights for the final output

The ANN models are complex by nature and trying to track and interpret the weight of each input or neuron may become challenging especially when there are multiple hidden layers and numerous input-variables. However, in our case we can start exploring each layer to get a better understanding of how each neuron is computed. In Figs. 5, 6, 7 and 8, the weight of each input/neuron is represented as a rectangle. The absolute value of the weight is directly proportional with the size of the rectangle and while filled rectangles represent negative weights, the empty rectangles represent positive weights.

It is important to mention that the output may depend more on how the information is reorganized inside the ANN model than it depends on the set of weights for the first layer of neurons. Figure 6 presents the weights of the first layer of neurons used to compute the second layer of neurons. Certain inputs may have bigger weights (positive/negative) in certain neurons and the weight of the neuron itself may vary in the ANN model.

By the third layer of neurons presented in Fig. 7, it can already be noticed that the weight of the intercept is significantly reduced compared to weights of intercept used to compute the first layer of neurons.

Finally, as described in Fig. 8, the intercept becomes almost not significant for computing the output. The second and third neurons have positive weights, while the first neuron has a negative weight in this process.

Instead of trying to track each weight and understanding how the information is organized in an ANN model, it is recommended to rely on the accuracy and relevancy of the model.

The results suggest that our model describes well the level of education by estimating it correctly with an accuracy of 95% on the training dataset. Moreover, it has a sharp predictive power managing to predict the level of education on a testing sample with an accuracy of 92% (Appendix S1). The Area Under the Curve (AUC) for the test sample is 0.86 (Appendix S2). Area under the Receiver Operating Characteristic (ROC) curve is one of the most important evaluation metrics for checking any classification model’s performance (Bradley 1997).

**Fig. 9:**
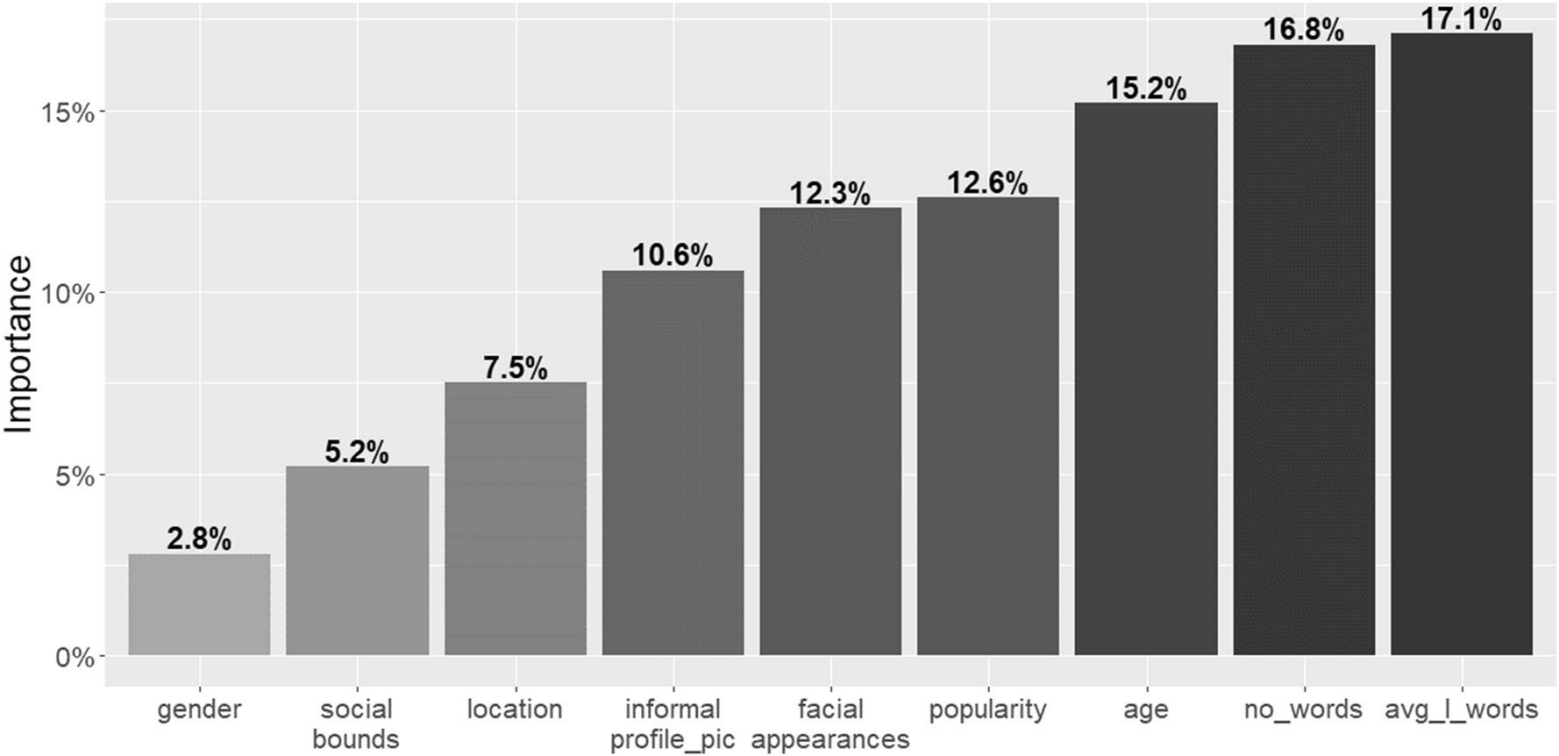
Relative importance coefficient of input-variables

Finally, following the novel method described in Sect. 3.3 we assess the importance of each variable. In line with our initial expectations, the results show that the most important variables for our ANN model are represented by the “average length of the words used” (RIC = 17%), the “number of words used” (RIC = 16.8%), and the “age” of the individuals (RIC = 15%). The second group of factors in term of importance are related to popularity and facial appearance, while location and social bounds are less relevant. The variable indicating the “gender” of the individuals has the lowest importance value (Fig. 9).

**Fig. 10:**
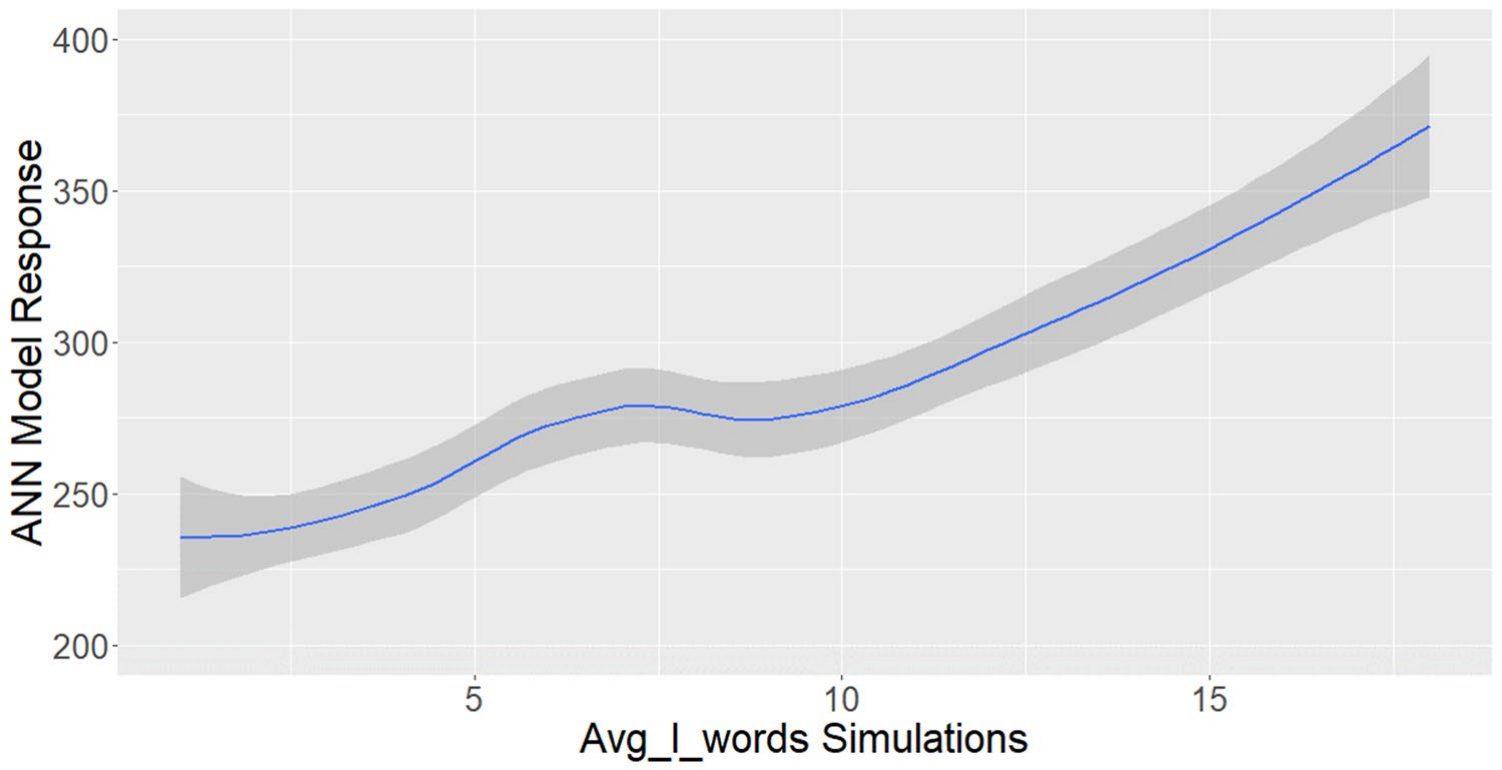
High-skilled probability maximization by word length

As described in Fig. 10, our model indicates that the probability to hold a position which requires tertiary education increases when the average word length increases. Nonlinear explanatory models can be developed to estimate more specifically by how much the probability increases when the average word length increases. However, this is out of our paper’s scope as we mainly aim to build a robust ANN model to predict if the individuals hold a job/position which requires tertiary education.

**Fig. 11:**
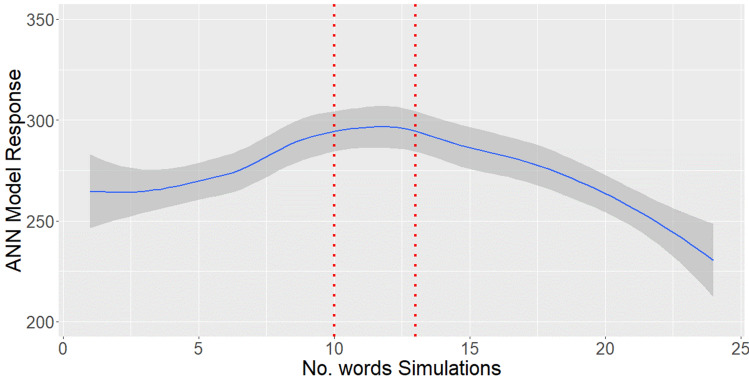
High-skilled probability maximization by number of words

Figure 11 indicates that the probability to hold a position which requires tertiary education increases when the number of words used ranges between ten and thirteen.

**Fig. 12:**
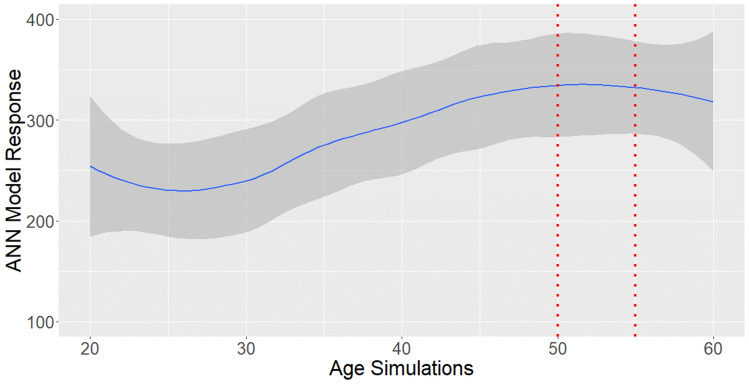
High-skilled probability maximization by age

As presented in Fig. 12, the “high-skilled” probability is also maximized when the age ranges between 50 and 55 years old.

Moreover, the model indicates that popularity is also an indication of individuals who are more likely to hold a position that requires tertiary education. The ANN model also indicates that a bigger regional high-skilled rate would generally increase this probability. On the other hand, the other variables are relevant only when the information is combined with the information from other variables as they did not change the number of predicted high-skilled persons significantly during the simulations (to not be confounded with the accuracy impact).

## Conclusions

Having the purpose to explore the education level of the Romanian Twitter users, this paper provides an innovative approach in terms of data used and applied method. It employed an alternative data-source, such as Social Media Platforms, and ANN and R programming for data collection and analysis. The model proposed used nine variables and three hidden layers of neurons to identify high-skilled users. Among the nine variables, we included the average length of the words and the number of words used by Romanian individuals to describe themselves.

The model describes well the level of education by estimating it correctly with an accuracy of 95% on the training dataset. Moreover, it has a sharp predictive power managing to predict the level of education on a testing sample with an accuracy of 92% and AUC of 0.86.

Our research also provided a clear and robust methodology for extracting explanatory insights from an ANN model and show that approximately 30% of users are highly skilled. Although our dataset contains more men than women, the percentage of high-skilled persons tends to be the same among the two genders. The percentage of high-skilled Twitter users tends to be lower among young users with age between 20 and 30. That may be explained by the minimum expected graduation age.

Employing a novel methodological approach, when evaluating the relevance of the inputs used in ANN models, the complexity of the phrases used in the self-descriptions by the Twitter users prove to be the most important.

This paper confirmed that social media data could provide relevant information for the study of the latest social patterns. It has also shown that Twitter data can provide valuable information at particular moments in time. The results of this paper may be used to go beyond understanding the details of the Romanian Twitter Users. The methods and the data developed and applied prove to be successful in predicting and explaining the education level in a sample, even if this is presented in non-structured manner. Therefore, the paper opens room for further research on the social characteristics and behavior of social media users.

## Supplementary Information

Below is the link to the electronic supplementary material.

Appendix S1.The prediction accuracy of the ANN model (TIFF 158 KB)

Appendix S2. Model’s performance (TIFF 12 KB)

## Footnotes

1 “At the beginning of the 1970s, Eurostat set up the NUTS classification as a single, coherent system for dividing up the EU's territory in order to produce regional statistics for the Community. For around thirty years, implementation and updating of the NUTS classification was managed under a series of "gentlemen's agreements" between the Member States and Eurostat.” (https://ec.europa.eu/eurostat/web/nuts/history).
