# Supplementary figures and images for: Artificial neural networks applied for predicting and explaining the education level of Twitter users
Source: Soc Netw Anal Min. 2021 Nov 1;11(1):112. doi: 10.1007/s13278-021-00832-1 (PMC8558764; doi:10.1007/s13278-021-00832-1)

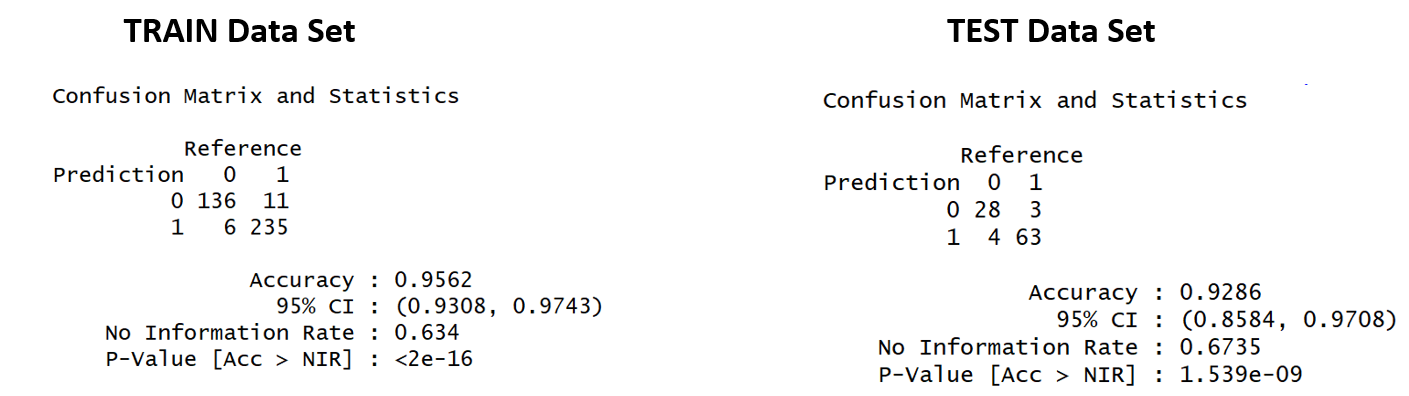

Supplement: Supplementary file 1 — Appendix S1.The prediction accuracy of the ANN model (TIFF 158 KB) [file 13278_2021_832_MOESM1_ESM.tiff]

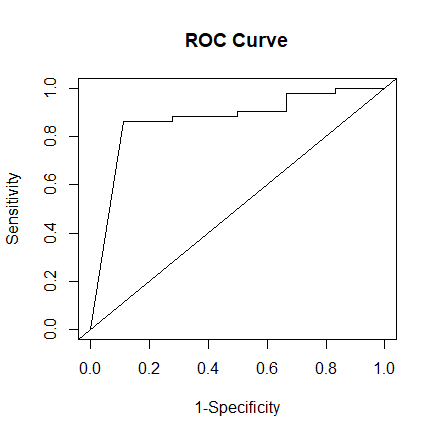

Supplement: Supplementary file 2 — Appendix S2. Model’s performance (TIFF 12 KB) [file 13278_2021_832_MOESM2_ESM.tiff]
